# Wearable Goniometer and Accelerometer Sensory Fusion for Knee Joint Angle Measurement in Daily Life

**DOI:** 10.3390/s151128435

**Published:** 2015-11-11

**Authors:** Alessandro Tognetti, Federico Lorussi, Nicola Carbonaro, Danilo de Rossi

**Affiliations:** 1 Research Center E.Piaggio, University of Pisa, Largo L. Lazzarino 1, 56126 Pisa, Italy; E-Mails: f.lorussi@ing.unipi.it (F.L.); nicola.carbonaro@centropiaggio.unipi.it (N.C.); d.derossi@centropiaggio.unipi.it (D.R.); 2 Information Engineering Department, University of Pisa, Via G. Caruso 16, 56122 Pisa, Italy

**Keywords:** wearable goniometers, accelerometers, data fusion, human motion analysis, joint angle measurements, knitted piezoresistive fabrics, smart textiles, sensor to segment alignment, knee joint

## Abstract

Human motion analysis is crucial for a wide range of applications and disciplines. The development and validation of low cost and unobtrusive sensing systems for ambulatory motion detection is still an open issue. Inertial measurement systems and e-textile sensors are emerging as potential technologies for daily life situations. We developed and conducted a preliminary evaluation of an innovative sensing concept that combines e-textiles and tri-axial accelerometers for ambulatory human motion analysis. Our sensory fusion method is based on a Kalman filter technique and combines the outputs of textile electrogoniometers and accelerometers without making any assumptions regarding the initial accelerometer position and orientation. We used our technique to measure the flexion-extension angle of the knee in different motion tasks (monopodalic flexions and walking at different velocities). The estimation technique was benchmarked against a commercial measurement system based on inertial measurement units and performed reliably for all of the various tasks (mean and standard deviation of the root mean square error of 1.96 and 0.96${}^{\circ }$, respectively). In addition, the method showed a notable improvement in angular estimation compared to the estimation derived by the textile goniometer and accelerometer considered separately. In future work, we will extend this method to more complex and multi-degree of freedom joints.

## 1. Introduction

Human posture and movement analysis is fundamental for a wide range of applications and disciplines, such as physical and neuro-rehabilitation, sports medicine, human performance assessment and virtual training. Although standard motion analysis instruments are widely used in these fields, the development and validation of ambulatory and unobtrusive sensing systems, which provide a reliable measurement of human motion and activity in out-of-lab daily life, are still open issues in the current literature. The development and validation of wearable technologies aimed at allowing physicians and therapists to remotely supervise and coach the patients during their rehabilitation exercise in their recovery phase are some of the current challenges.

Recently, as anticipated by De Rossi and Veltink in [1], wearable textile-integrated sensing of human movements and electrophysiological acquisition devices have been developed. The authors of the current paper collaborated, within the EU project INTERACTION [2], in the development of an e-textile platform endowed with wireless 3D inertial movement and textile-based stretch and goniometric sensing [3]. The INTERACTION system was tested in post-stroke patients to assess the quality of their mobility and their reaching and grasping capacities.

Over the last decade, inertial measurement units (IMUs), based on the integration of accelerometers, gyroscopes and magnetometers, have gained the reputation of being at the cutting edge of wearable motion tracking [4,5,6,7,8]. IMUs estimate the orientation of the body segments where they are attached by combining multi-sensor information through dedicated optimal sensor fusion algorithms mainly based on Kalman filtering [4,5,7]. The general approach is to apply strap-down integration of the gyroscope signal [9] and to correct the inclination and heading drifts through the accelerometer and magnetometer measurements. The combination of different IMUs, placed on connected body segments, and the additional information on the kinematic constraints enable joint angles to be measured [10,11]. IMU-based systems can also be employed to track the velocity of the human body, as demonstrated in the study performed by Yuan [12].

Several wearable motion analysis systems based on IMU technology are now on the market. The well-known XSens MVN system [13,14] uses proprietary wireless motion trackers (MTw) applied to an adherent suit to perform full-body motion tracking (upper/lower limbs, trunk and neck). Each MTw includes a tri-axial accelerometer, a triaxial gyroscope and a tri-axial magnetometer and computes the fusion algorithms to calculate the global orientation of the body segment to which it is attached. The orientations of the single body segments are further fused by means of proprietary bio-mechanical models to estimate the full kinematics of the human body. XSens systems, which were used in the current study to benchmark the proposed technique, have been adopted and tested in many studies. For example, Zhang [15] showed a mean error below two degrees by comparing the knee flexion-extension detected by two MTws with the same angle retrieved by a stereophotogrammetric system. Similar IMU-based systems focusing on gait analysis [16,17] have been marketed by INSENCO Co. (composed of nine inertial sensors placed on the lower limbs combined with wireless six-axis force sensors [18]) and by Tec Gihan (the M3D system that combines inertial measurement units and wearable force plates [19]).

Beside their widespread adoption in the field, IMU-based systems suffer from a loss of accuracy due to magnetic disturbance; they can be bulky, and their cost can be considerable. The contribution of inertial and translational accelerations can limit the accuracy of the accelerometer update [20]. Most importantly, the presence of ferromagnetic materials or other magnetic disturbances due to environmental noise, which is inevitable during daily life applications, limits the accuracy of the heading compensation [21,22] and can compromise the full orientation estimation. Considering our context of wearable unobtrusive sensing in daily life, an ambulatory, yet reliable monitoring system that avoids the use of magnetometers would be beneficial, as already pointed out by Seel in [23].

Thus, the new approach described in this work exploits only the acceleration components of IMUs in combination with other measurement sources taken from a particular class of e-textile sensors.

Textile-based or e-textile solutions have been developed, and their possible application for ambulatory and unobtrusive motion detection has been described in several research papers, including [24,25,26,27,28]. Textile-based solutions have several advantages compared to solid-state sensors: low cost, lightweight, low thickness, flexibility and the possibility of adaptation to different body structures. By exploiting these kinds of sensors, it is possible to design sensing garments with sensor strips applied to specific locations on normal cloth. Despite these attractive characteristics, textile sensor adoption is still limited mainly due the low reliability, which thus limits their use to the reconstruction of large and slow movements. We recently developed a new generation of textile-based goniometers, obtained by coupling two layers of knitted piezoresistive fabrics (KPF) through an electrically-insulated layer, as described in [29]. Compared to previously-developed e-textile sensors, textile goniometers can provide an accurate and reliable measurement of the angles between connected body segments [29,30,31]. Although textile goniometers represent an important step forward in wearable human motion detection, they still have their limitations. In particular, the sensor calibration, which has to be performed in two known angular positions, as described in Section 2.1, needs to be computed once the sensor has been integrated into the textile and worn by the user. Due to the intrinsic nature of the textile materials, which tend to modify their mechanical properties over time, the calibration coefficients can slowly drift, and the calibration procedure needs to be repeated periodically. This is highly undesirable in ambulatory and daily life applications where the subject has to use the system as is, without performing too many operations for the system to work properly. Moreover, we pointed out in [29] that although KPF goniometers represent a consistent step forward in motion sensing through e-textiles, a slight hysteresis can still affect the sensor performance.

The aim of this paper is to show that by combining e-textiles and low cost inertial sensing, unobtrusive and reliable human motion monitoring is possible. This can be achieved by addressing the drawbacks of the two measurements, by fusing the information derived from KPF goniometers and tri-axial accelerometers. The idea behind this is that single triaxial accelerometers are less expensive and complex compared to the current complete IMU technology and do not suffer from magnetic interference. In addition, they can be easily attached or integrated into the extremities of the KPF goniometer in order to obtain a reliable and unobtrusive measurement system.

Without loss of generality, we demonstrated our concept on the movement of the femoral-tibial joint, which along with the hip and ankle is one of the three major articulations of the lower limb that allows ambulation.

Several research groups have investigated performing reliable knee joint kinematic assessments through simplified and reduced sensing systems based on accelerometers and gyroscopes and avoiding the use of the magnetometer information. Relevant studies are reported in [23,32,33,34,35,36,37] and exploit the inertial information derived by two accelerometer/gyroscope pairs that are attached to the thigh and shank segments. In most of the cited publications, the reconstruction technique is based on the estimation of thigh and shank orientation to calculate the knee flexion-extension angle [33,35,36]. The accuracy of these techniques relies on the precision of the orientation estimation, which is a challenge due to the absence of the magnetometer measurement, and the good alignment of the accelerometer/gyroscope reference frames with respect to the body segment reference frames. Significant results, root mean square error (RMSE) around two degrees in comparison with an optical system, have been obtained by Favre [33] through an alignment procedure that exploited a static standing posture and predefined hip abduction/adduction movements. Another interesting work was recently presented by Seel in [23], who proposed a new method for joint axis identification that does not rely on the accuracy with which the subject performs predefined postures or movements, as well as an innovative reconstruction technique. Their technique is based on the integration of the angular rate on the identified joint axis and the subsequent correction of the slowly-drifting angle by the accelerometer measurement (RMSE of three degrees in comparison with an optical reference system).

In this work, we propose an innovative solution for the implementation of a simplified and reduced sensing system for knee joint flexion-extension assessment. We exploit a hybrid system that combines the non-inertial angular measurement of the textile goniometer with the inertial information derived from the two accelerometers. More specifically, we developed and conducted a preliminary evaluation of a dedicated sensory fusion approach, based on a Kalman filter implementation. This method exploits the accelerometer measurement to correct the angular estimation performed by the KPF goniometer by continuously adjusting its calibration parameters. We demonstrated the capabilities of the new hybrid measurement system, which is made up of KPF goniometers and tri-axial accelerometers, through the experimental setup described in Section 2. We built a sensing knee band prototype with pockets used to host a KPF goniometer, which detects the angle between femur and tibial segments, and two IMUs attached to the the thigh and shank segments. Raw accelerometer data and KPF outputs were processed through the fusion technique (see Section 3) to estimate the knee flexion-extension angle. At the same time, the whole information set derived from the thigh and shank IMUs (*i.e*., the full orientation matrix extracted by fusing accelerometer, gyroscope and magnetometer information) was employed as the standard reference for benchmarking our new approach. In the experimental session, the prototype was employed to acquire goniometer and IMU data while subjects performed different tasks involving moving their knee joints. To introduce the sensing fusion technique, the KPF device and inertial sensing system working principles are described in Section 2.1 and Section 2.2, together with the calibrations of the two systems needed to start the experimentation. The calibration procedure in Section 2.1, which identifies the parameters of the KPF goniometer, is fundamental to determine the initial status of the Kalman filter procedure described in Section 3. The main part of the paper describes the algorithm that improves the quality of the knee position estimation, by refining the goniometer parameters. Finally, in Section 4, the knee flexion-extension estimate performed by the hybrid sensing system, detected in six different movement tasks, is compared to the reference measurement system to evaluate its performance. In the whole set of tests, the fusion technique showed a reliable performance and was effective at measuring dynamic knee movements (across trials, mean and standard deviation of the RMSE equal to 1.96 and 0.96 degrees, respectively). These results make our approach comparable with other ambulatory measurement systems. The results also demonstrated that the fusion method significantly improved the measurement performance of the individual sensor classes (accelerometers and goniometers considered individually *vs*. the reference measurements). In addition, note that no hypotheses were made on the accelerometer position and orientation with respect to the joint reference frame. Note also that the system can be easily calibrated, through the dedicated procedure conceived of within this work, without requiring the user to perform complex tasks. These achievements and the promising measurement performance make the hybrid system a good candidate for ambulatory and unobtrusive wearable measurement systems.

## 2. Experimental Setup

**Figure 1:**
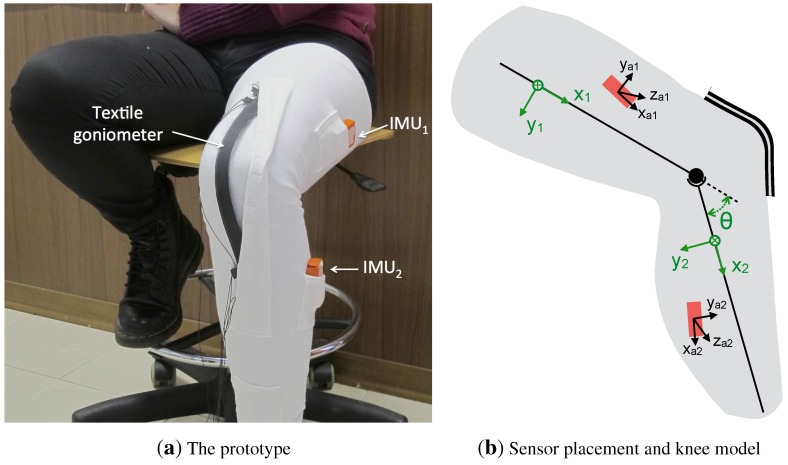
Sensing prototype and sensor placement around the knee joint. (**a**) A double-layer knitted piezoresistive fabrics (KPF) goniometer and two inertial measurement units (IMUs) were applied to the knee band; (**b**) A geometrical scheme of the reference frames fixed with the body segments and the IMUs. The knee was simply modeled as a hinge joint, and the flexion-extension angle (*θ*) was defined as the angle between the two consecutive model segments (*i.e*., the angle between the *x* unit vectors of the $\Psi_{1}\left[x_{1},y_{1},z_{1}\right]$ and $\Psi_{2}\left[x_{1},y_{1},z_{1}\right]$ frames). IMU and accelerometer reference frames ($\Psi_{a_{1}}\left[x_{a_{1}},y_{a_{1}},z_{a_{1}}\right]$ and $\Psi_{a_{2}}\left[x_{a_{2}},y_{a_{2}},z_{a_{2}}\right]$) are not aligned with the corresponding segment reference frame.

In order to acquire data concerning the flexion-extension of the femoral-tibial joint, both from KPF goniometers and inertial sensors, a knee band prototype was created using a Lycra fabric. The band was specifically designed with pockets to contain the KPF and inertial sensing, as shown in Figure 1a. As shown in in Figure 1b, the knee was simply modeled as a hinge joint, and the flexion-extension angle (*θ*) was defined as the angle between the two consecutive segments of the model (*i.e*., the angle between the *x* unit vectors of the $\Psi_{1}\left[x_{1},y_{1},z_{1}\right]$ and $\Psi_{2}\left[x_{1},y_{1},z_{1}\right]$ frames).

The KPF goniometer (length 30 cm, width 2.5 cm and thickness 1.5 mm) was integrated into the garment to entirely cover the considered joint. The working principle of the goniometer, produced by SMARTEX [38] and based on our proprietary design, is reported in Section 2.1. The goniometer was calibrated before being integrated into the knee band according to the procedure described in Section 2.1.

In the same knee band, two inertial measurement units (MTw by XSens, [13]) were fixed inside the pockets at the thigh ($IMU_{1}$) and the shank ($IMU_{2}$). IMUs are positioned on the knee band without any assumptions regarding their orientation and position with respect to the knee joint segments. The XSens MTws [13] used in the experiments are compact IMUs containing 3D linear accelerometers, 3D rate gyroscopes and 3D magnetometers. Each MTw performs real-time signal elaboration and transmits, through a wireless link, the 3D orientation (Euler, quaternions or rotation matrices) and raw sensor data (acceleration, angular velocities, Earth magnetic field orientation). The raw accelerometer component available from the IMU signal was used in combination with the textile goniometer, as detailed in Section 3, hereafter named the accelerometer sensor. The knee flexion-extension angle derived from the IMU outputs can be computed by taking into account the components of the full rotation matrix, which describes the orientation of the IMU frame on the shank with respect to the frame of the IMU placed on the thigh [10,14]. The IMU devices were calibrated according to the procedure provided by the producer [14]. The resulting knee flexion extension angle ($\theta_{r}$) was considered as the reference measurement to evaluate the textile goniometer/accelerometer hybrid system that we propose in this work.

In order to collect data on the knee kinematics, in the experimental sessions, the band was worn by five healthy subjects, who were asked to perform several tasks, grouped into a set of six trials and repeated three times. In the first two trials, the subjects were asked to perform knee flexion-extension movements at different velocities in monopodalic contralateral standing. During these phases, the subjects were asked to flex/extend the knee approximately in the 0–$90^{\circ }$ range. The last four trials concerned walking activities with increasing velocities. The subjects were asked to walk freely on a straight line for about 40 s, starting with slow-speed walking (first trial) up to very fast walking (fourth trial). The subjects were free to select the velocities without external constraints. Before starting to collect the full set of data, each subject was asked to perform the simple movements necessary to align the thigh and shank accelerometer frames following the calibration protocol described in Section 2.2.

KPF goniometer output was gathered through a dedicated wireless electronic unit, and data were sampled at 100 Hz and transmitted to a remote PC for elaboration. The IMU sampling rate was set to 100 Hz, and the data (raw accelerometer outputs and orientation expressed as the rotation matrix) were transmitted via a wireless link to the same PC. IMU and goniometer data were synchronized through a dedicated XSens software tool. After recording the sensor outputs, our algorithm for the estimation of the knee flexion-extension angle, which fuses data from the KPF goniometer and the accelerometers (described in Section 3), runs offline on a remote PC. The knee flexion-extension angle (*θ*), defined in Figure 1b, was extracted by the fusion method applied to goniometer and accelerometer outputs with the same update rate of the input signals (100 Hz).

### 2.1. KPF Goniometers

**Figure 2:**
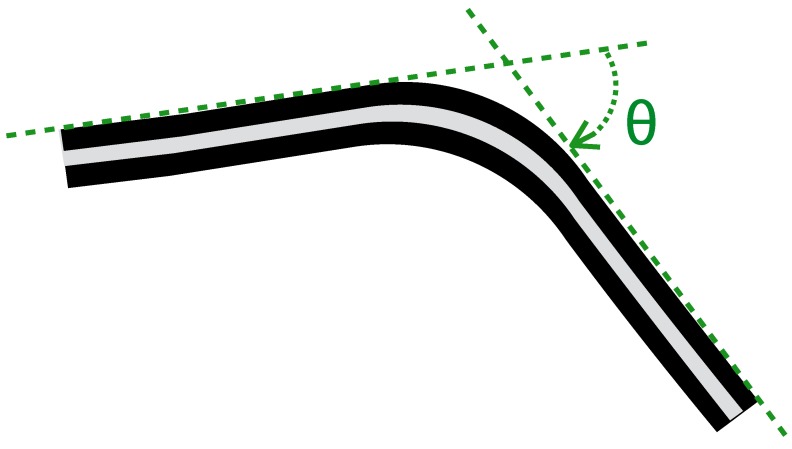
Schematic diagram of a double-layer KPF goniometer. The black stripes represent the two identical piezoresistive layers, while the gray stripe is the insulating layer. When the sensor is in the flat position, the resistance difference (ΔR) between the two layers is zero. When the sensor is flexed, ΔR is proportional to the bending angle (*θ*), defined as the angle between the tangent planes to the sensor extremities (green dashed line in the picture).

This section briefly reports the background and the working principle of the KPF goniometer used in our experiments. As described in our previous studies [29,30,31] and reported in Figure 2, our textile goniometers were developed by coupling two piezoresistive layers through an electrically-insulating layer. The sensing layers were made of knitted piezoresistive fabrics (KPFs), previously employed as single-layer strain sensors for monitoring biomechanical and cardio-pulmonary parameters [39,40].

Ideally, if the two KPF layers were geometrically and electrically equivalent, the sensor output, represented by the resistance difference between the two sensing layers (ΔR), vanishes when the device is flat and is proportional to the flexion angle (*θ*), except for a second order infinitesimal function [29]:

(1) ΔR=kθ

In practice, Equation (1) is not verified, due to the differences in the electrical properties between the two piezoresistive layers. In [29], the *θ* *vs*. ΔR relation can be reasonably approximated by the following linear function:

(2) $$\Delta R=s_{\theta }\;\theta +\Delta R_{o}$$

where $s_{\theta }$ and $\Delta R_{0}$ represent the goniometer sensitivity and offset, respectively. The angle values *θ* can be explained by Equation (2) as:

(3) $$\theta =\frac{\Delta R-\Delta R_{o}}{s_{\theta }}=c_{1}\;\Delta R+c_{2}$$

In Equation (3), parameters $c_{1}$ and $c_{2}$ remain unknown, and it is necessary to perform an initial calibration to determine them. Note that $c_{1}$ and $c_{2}$ are the process variables in the Kalman filtering described in Section 3.1 and that their initial estimation is thus the zero status for the iterative process of data refinement. According to Equation (3), by acquiring $\Delta R_{1}$ and $\Delta R_{2}$ in two different angular positions $\theta_{1}$ and $\theta_{2}$, it is possible to compute $c_{1}$, $c_{2}$ as:

(4) $$\left\{\begin{array}{c} c_{1}=c_{1,0}=\frac{\theta_{1}-\theta_{2}}{\Delta R_{1}-\Delta R_{2}}\\ c_{2}=c_{2,0}=\frac{\Delta R_{1}\;\theta_{2}-\Delta R_{2}\;\theta_{1}}{\Delta R_{1}-\Delta R_{2}} \end{array}\right.$$

Before starting to collect the data in our trials, the goniometer was calibrated in two angular positions ($\theta_{1}=0^{\circ }$ and $\theta_{2}=90^{\circ }$) to obtain the $c_{1,0}$ and the $c_{2,0}$ values to feed the Kalman cycle.

### 2.2. Accelerometer Alignment

The data fusion algorithm, which is the core of the current research and is described in Section 3, combines the information from the KPF goniometer and the triaxial accelerometers fixed on the thigh and shank (raw accelerometer components of the IMU signal). Considering the unknown and unmeasured orientation and the position of the accelerometers with respect to the knee joint segments (as shown in Figure 1a,b, the accelerometer frames need to be aligned with the corresponding joint segments ($\Psi_{a_{1}}\to \Psi_{1}$,$\Psi_{a_{2}}\to \Psi_{2}$).

**Figure 3:**
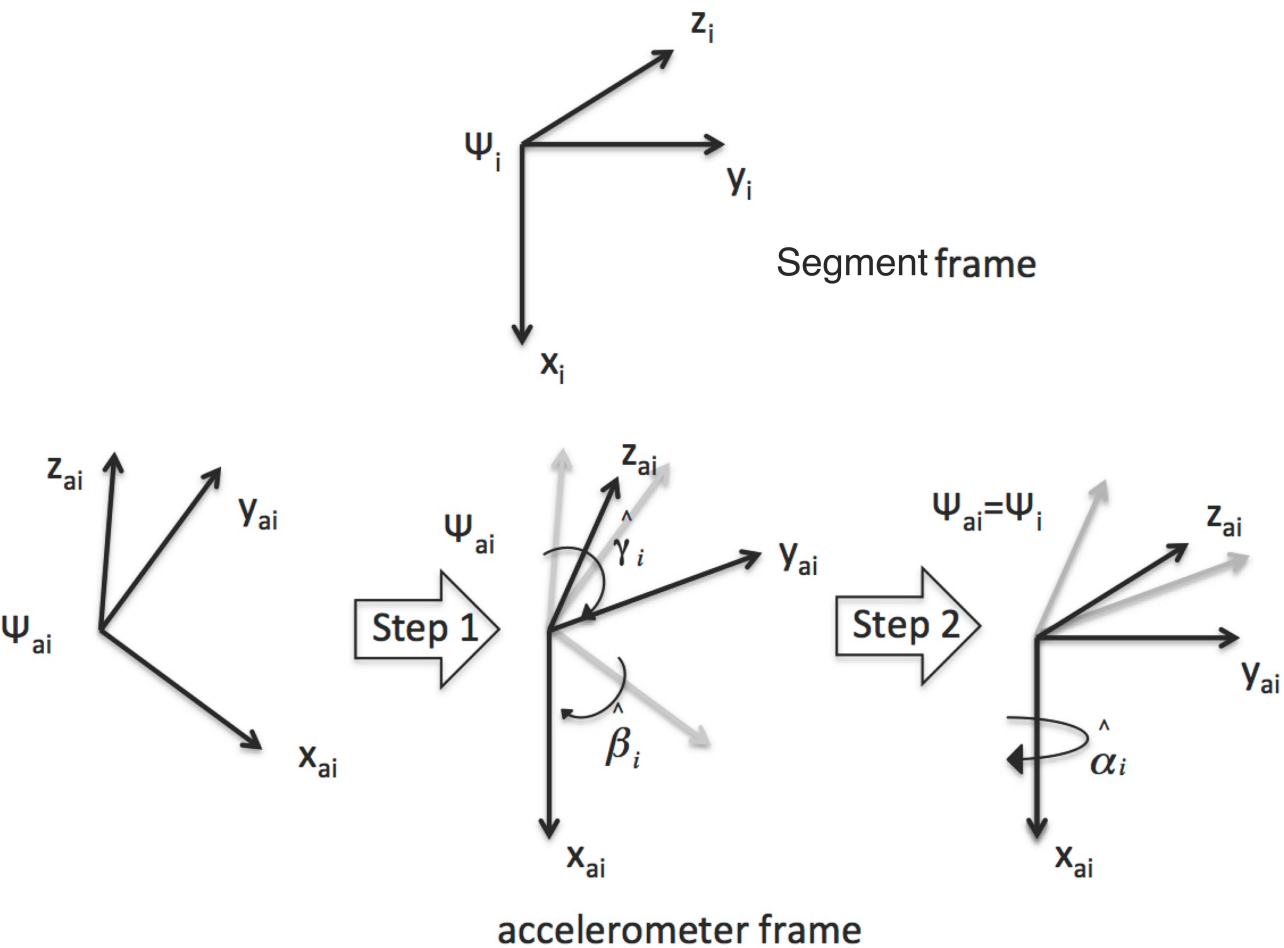
The calibration procedure for the accelerometers. In the first step, using the accelerometer output only, acquired from a subject in standing position, the $x_{ai}$ axes of the accelerometer frame are aligned with the corresponding $x_{i}$ axis of the bone frames by computing the ${\hat{\gamma }}_{i}$ and ${\hat{\beta }}_{i}$ angles. In the second step, using data collected by the goniometer and the accelerometers in a dynamic acquisition, the remaining axes of the inertial frames are aligned with the corresponding axes of the frames fixed with the joint segments.

For this reason, we designed the sensor-to-segment calibration procedure outlined in Figure 3. The required re-orientation of the accelerometer frames can be obtained as an accelerometer calibration protocol, which is performed on-body and requires knowledge of the goniometer output. The only assumption is that the position/orientation of the accelerometer frames with respect to the joint segments remains constant (*i.e.*, we neglect muscle artifacts that could otherwise be reduced with a strategic positioning of the sensors).

In the first step, using the accelerometer output in a static position, it is possible to determine the transformation that maps the $x_{ai}$, i=1,2 axes of the two frames into the corresponding axes of the segment frames. This transformation is performed by computing the ${\hat{\gamma }}_{i}$ and ${\hat{\beta }}_{i}$ angles that can be obtained from the output vector of the uncalibrated accelerometer $\eta_{i}$, as:

(5) $$\left\{\begin{array}{c} {\hat{\gamma }}_{i}=\arctan\left(\frac{-\eta_{ix}}{\sqrt{\eta_{iy}^{2}}+\eta_{iz}^{2}}\right)\\ {\hat{\beta }}_{i}=\arctan\left(\frac{\eta_{iy}}{\eta_{iz}}\right) \end{array}\right.$$

To gather static information from the accelerometers, the subject was asked to stand upright in a natural position. In this position, the $x_{i}$’s are aligned with the gravity vector, except for the physiological angles of the leg bones in standing position, which are negligible with respect to the range of motion of the femoral-tibial joint. In the second step of the alignment procedure, starting from the upright position, the subject was asked to slowly flex the knee three times (in monopodalic standing). Let us suppose that the movement is spanned by a time variable k∈[0,N]. Then, the angles ${\hat{\alpha }}_{1}$ and ${\hat{\alpha }}_{2}$, necessary to complete the frame re-orientation, are computed as follows:

(6) $$\left({\hat{\alpha }}_{1},\;{\hat{\alpha }}_{2}\right)=\underset{\alpha_{1},\alpha_{2}}{arg\;min}\sum_{k=0}^{N}{∥R_{x}\left(\alpha_{2}\right)\;\eta_{2}^{*}-R_{21}\left(\theta_{g}\right)\;R_{x}\left(\alpha_{1}\right)\;\eta_{1}^{*}∥}_{2}$$

where:

$$R_{x}\left(\alpha_{i}\right)=\left(\begin{array}{ccc} \cos\;\;(\alpha_{i}) & \sin\;\;(\alpha_{i}) & 0\\ -\sin\;\;(\alpha_{i}) & \cos\;\;(\alpha_{i}) & 0\\ 0 & 0 & 1 \end{array}\right)$$

describes a rotation around the $x_{i}$, i=1,2 axis, $\eta_{i}^{*}$ are the accelerometer outputs after the first calibration step, $\theta_{g}$ is the flexion angle obtained by the nominal goniometer output and $R_{21}\left(\theta_{g}\right)$ is the rotation of the angle detected by the goniometer along the knee anatomical rotation axis (parallel to $z_{i}$ in Figure 1b). The calibrated accelerometer outputs ($\eta_{acci}$) are then obtained by simply rotating $\eta_{i}^{*}$ by the corresponding rotation matrix $R_{x}({\hat{\alpha }}_{i})$.

To test the reliability of the accelerometer calibration procedure, let us introduce the following metric:

(7) $$mis_{S}\left(R\right)={∥R\;S^{T}-I∥}_{2}$$

Where R, S are a special orthogonal matrix and I the identity in $R^{3\times 3}$. The misalignment function $mis_{S}:SO3\to R^{+}$ is a real map defined on orthogonal frames (the columns of *R*), and it is possible to prove, thanks to the triangle inequality, that its values lie in the range [0,2]. For $mis_{S}\left(R\right)={∥R\;S^{T}-I∥}_{2}=0$, matrices *R* and *S* are completely aligned, while for $mis_{S}\left(R\right)=2$, matrices *R* and *S* are “intuitively” completely misaligned. Function $mis_{S}$ provides an indication of the reliability of the presented calibration method for the accelerometers. In particular, we adopted the $mis_{S}$ metric to compare the accelerometer sensor-to-segment calibration matrices with the corresponding IMU calibration matrices obtained by the standard procedure provided by the producer. Below is the result of the alignment for the accelerometer and the IMU frame placed on the shank. The sensor-to-segment calibration matrix derived from the XSens MTw is given by:

(8) $$S=\left(\begin{array}{ccc} 0.9770 & 0.0313 & -0.2108\\ -0.0221 & 0.9987 & 0.0462\\ 0.2120 & -0.0405 & 0.9764 \end{array}\right)$$

While the accelerometer calibration matrix derived by the method described above and built using (${\hat{\alpha }}_{2},\;{\hat{\beta }}_{2},\;{\hat{\gamma }}_{2}$) holds:

(9) $$R=\left(\begin{array}{ccc} 0.9766 & 0.0356 & -0.2120\\ -0.0224 & 0.9977 & 0.0645\\ 0.2138 & -0.0583 & 0.9751 \end{array}\right)$$

Since IMUs and accelerometers refer to the same reference frame, the misalignment before calibration can be determined considering, in the $mis_{S}$ map, no transformations applied to the accelerometer signal (*i.e*., R=I) and the same IMU sensor-to-segment calibration matrix S. In this case, the misalignment function holds:

(10) $$mis_{S}\left(I\right)={∥I\;S^{T}-I∥}_{2}=0.214$$

While using the matrix *R* generated in the calibration procedure, we obtain:

(11) $$mis_{S}\left(R\right)={∥R\;S^{T}-I∥}_{2}=0.016$$

Which ensures that $\Psi_{2}$ and $\Psi_{a2}$ are, in practice, aligned. Similar results were obtained for the accelerometer alignment across the whole set of trials carried out in this work.

## 3. Fusion Algorithm

**Figure 4:**
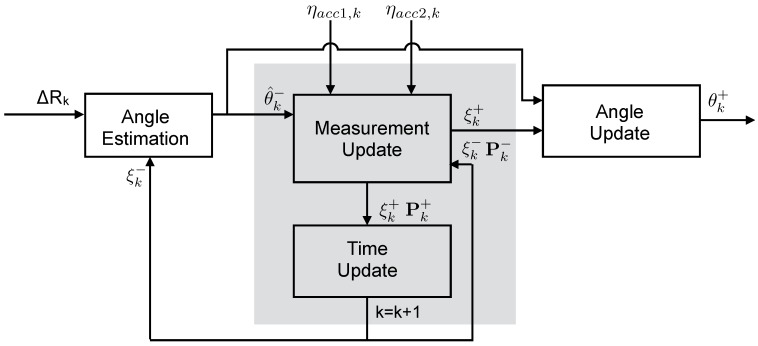
The goniometer/accelerometer fusion methods. The grey box represents the Kalman filter in its error state or indirect form.

To estimate the knee flexion extension angle by fusing the information of the KPF goniometer and the thigh and shank accelerometers, a dedicated Kalman filter was developed. The knee flexion-extension (*θ*) was defined as the angle between the *x* unitary vectors of the reference frames of the hinge joint segments ($\Psi_{1}$ and $\Psi_{2}$, defined in Figure 1b). The filter is used to correct the goniometer angular estimation through a measurement update performed on the accelerometer data and exploiting the knowledge of the joint kinematic constraints. As shown in Figure 4, the goniometer nominal calibration parameters, defined in Equation (3), are continuously updated according to the filter outputs. To efficiently correct the goniometer output by means of the accelerometer information, the accelerometer reference frames ($\Psi_{a1}$ and $\Psi_{a2}$, shown in Figure 1b) have to be aligned with the frames defined for the knee joint model ($\Psi_{1}$ and $\Psi_{2}$), as reported in the accelerometer calibration procedure in the previous section.

### 3.1. Estimation Procedure

At each time sample *k*, the *a priori* angular estimation ${\hat{\theta }}_{k}^{-}$ is performed taking into account the goniometer output and the *a priori* estimation of the calibration parameters (${\hat{c}}_{1,k}^{-}$, ${\hat{c}}_{2,k}^{-}$):

(12) $${\hat{\theta }}_{k}^{-}={\hat{c}}_{1,k}^{-}\;\Delta R_{k}+{\hat{c}}_{2,k}^{-}$$

The *a priori* estimation of the knee flexion-extension is then fed into the measurement update stage, which introduces a correction by exploiting the thigh and shank accelerometer measurements ($\eta_{acc1,k}$, $\eta_{acc2,k}$). In the current description, $\eta_{acc1,k}$ and $\eta_{acc2,k}$ represent the signals of the accelerometers that have been aligned with the joint segment frames ($\Psi_{1}$, $\Psi_{2}$), as described in Section 2.2.

The Kalman filter developed within this study (see the gray box in Figure 4) is in the error state form (or indirect form) [41,42] and operates on the error of the actual state. This method has been proven to be a valid tool in the reconstruction of body kinematics with inertial sensors [43,44,45]. The filter employs the state space representation reported in the following equations:

(13) $$\xi_{k+1}=A\;\xi_{k}+\mu$$

(14) $$\zeta_{k}=C\;\xi_{k}+\nu$$

where *ξ* is the state vector to be estimated, *ζ* the observation vector, A and C represent the state transition and observation matrices and *μ* and *ν* are Gaussian processes and measurement noise (*i.e*., zero mean white noise processes). The state vector (*ξ*) is defined as follows:

(15) $$\xi_{k}={\left[\delta c_{1,k}\;\;\delta c_{2,k}\right]}^{T}$$

where $\delta c_{1,k}$ and $\delta c_{2,k}$ are the calibration parameter errors that can be defined by the difference between the true angle ($\theta_{k}$) and the actual estimation (${\hat{\theta }}_{k}$):

(16) $$\theta_{k}-{\hat{\theta }}_{k}=\delta \theta_{k}=\left(c_{1,k}-{\hat{c}}_{1,k}\right)\;\Delta R_{k}+\left(c_{2,k}-{\hat{c}}_{2,k}\right)=\delta c_{1,k}1\;\Delta R_{k}+\delta c_{2,k}\;$$

In the initialization phase, ${\hat{c}}_{1,0}^{-}$ and ${\hat{c}}_{2,0}^{-}$ are assigned with the nominal calibration values defined in Section 2.1. Then, at each time iteration, the filter processes the goniometer and the accelerometer signals and performs the measurement update, the angle update and the time update.

In the measurement update, according to the Kalman filter theory [46], the *a posteriori* (*i.e*., corrected) state and its covariance are calculated as follows:

(17) $$\xi_{k}^{+}=\xi_{k}^{-}+K_{k}\;\left(\zeta_{k}-C\;\xi_{k}^{-}\right)$$

(18) $$P_{k}^{+}=P_{k}^{-}-K_{k}\;C\;P_{k}^{-}$$

(19) $$K_{k}=P_{k}^{-}\;C^{T}{\left(C\;P_{k}^{-}\;C^{T}+R\right)}^{-1}$$

where $K_{k}$ represents the Kalman gain, R the measurement uncertainty (expressed by the covariance of the measurement noise *ν*) and minus and plus symbols indicate *a priori* and *a posteriori* estimates, respectively.

In the angle update phase, the calibration parameters and the angular estimation are corrected taking into account the measurement update results:

(20) $${\hat{c}}_{i,k}^{+}={\hat{c}}_{i,k}^{-}+\delta c_{i,k}$$

(21) $${\hat{\theta }}_{k}^{+}={\hat{\theta }}_{k}^{-}+\delta \theta_{k}={\hat{c}}_{1,k}^{+}\;\Delta R_{k}+{\hat{c}}_{2,k}^{+}$$

According to the indirect formulation of the Kalman filter [44,45], in the time update, the error states are set to zero, and the corresponding covariance is propagated through the state transition matrix (A) and the process noise covariance (Q):

(22) $$P_{k+1}^{-}=A\;P_{k}^{+}\;A^{T}+Q$$

To define the process model, we simply supposed that the calibration parameter errors were random walk processes, and consequently, the state transition matrix (A) was the identity matrix.

In the indirect formulation, the observation (*ζ*) is defined as the difference between measured and estimated sensor input. Following [45], we constructed the observation vector on the assumption that, if the two joint segments experience the same inertial acceleration, the difference between $\eta_{acc2,k}$ and its estimation ${\hat{\eta }}_{acc2,k}$ can be expressed as:

(23) $$\zeta_{k}=\eta_{acc2,k}-{\hat{\eta }}_{acc2,k}=\eta_{acc2,k}-{\hat{R}}_{21}\left(\theta_{k}\right)\;\eta_{acc1,k}$$

where ${\hat{R}}_{21}$ is the estimation of the rotation matrix that describes the transformation from the upper leg reference frame ($\Psi_{1}$) to the lower leg reference frame ($\Psi_{2}$). As reported in [45], Equation (23) is valid if the rotational acceleration contribution is sufficiently small. This condition can be tested from the difference in magnitude of the two accelerometer outputs, and the measurement covariance can be changed accordingly. Taking into account the constraints given by the hinge joint model, ${\hat{R}}_{21}$ can be expressed as a function of the *a priori* angular estimation:

(24) $${\hat{R}}_{21}\left(\theta_{k}^{-}\right)=\left(\begin{array}{ccc} \cos\;\;({\hat{\theta }}_{k}^{-}) & \sin\;\;({\hat{\theta }}_{k}^{-}) & 0\\ -\sin\;\;({\hat{\theta }}_{k}^{-}) & \cos\;\;({\hat{\theta }}_{k}^{-}) & 0\\ 0 & 0 & 1 \end{array}\right)$$

Finally, we obtained the observation matrix C by expressing $\zeta_{k}$ as a function of the state vector ($\xi_{k}$). We started from the observation that the true rotation matrix can be expressed as a function of ${\hat{R}}_{21}$ and δθ:

(25) $$R_{21}={\hat{R}}_{21}\;\left(I+\delta R\right)$$

where δR is function of $\delta \theta =\theta -\hat{\theta }$ and, for small δθ, can be approximated as follows:

(26) $$\delta R=\left(\begin{array}{ccc} \cos\;\left(\delta \theta \right)-1 & \sin\;\left(\delta \theta \right) & 0\\ -\sin\;\left(\delta \theta \right) & \cos\;\left(\delta \theta \right)-1 & 0\\ 0 & 0 & 0 \end{array}\right)\approx \left(\begin{array}{ccc} 0 & \delta \theta & 0\\ -\delta \theta & 0 & 0\\ 0 & 0 & 0 \end{array}\right)$$

Considering Equation (23) and Equation (25), the observation vector can be written as:

$$\zeta_{k}=R_{21}\;\eta_{acc1,k}-{\hat{R}}_{21}\;\eta_{acc1,k}=$$

(27) $$={\hat{R}}_{21}\;\left(I+\delta R\right)\;\eta_{acc1,k}-{\hat{R}}_{21}\;\eta_{acc1,k}=\delta R\;\eta_{acc1,k}=\left(\begin{array}{ccc} 0 & \delta \theta & 0\\ -\delta \theta & 0 & 0\\ 0 & 0 & 0 \end{array}\right)\;\eta_{acc1,k}$$

We then obtained the observation vector as a function of the state by taking into account Equation (27) and substituting Equation (16) for δθ.

## 4. Results

In this section, the knee flexion extension angle estimated by the hybrid system, obtained by fusing goniometer and accelerometer data as described in Section 3, is compared to the output of the reference measurement system (knee flexion extension angle obtained by the IMUs). In addition, to highlight the improvement of our fusion technique, a comparison between the reliability of our method (hybrid system *vs*. IMUs) and the errors committed by the individual sensors is presented (*i.e.*, accelerometers *vs*. IMUs and textile goniometer *vs*. IMUs).

**Figure 5:**
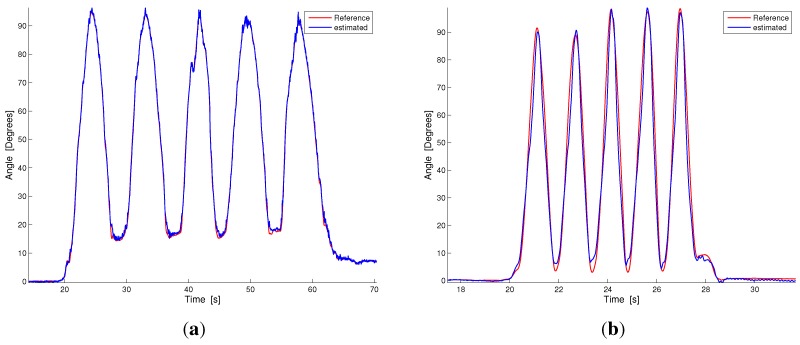
Dynamic comparison between our estimation technique and the reference measurement during contralateral monopodalic standing tasks with different knee flexion-extension velocities. The blue line represents our estimation, while the red line is the reference measurement. (**a**) Slow knee flexion; (**b**) Fast knee flexion.

**Figure 6:**
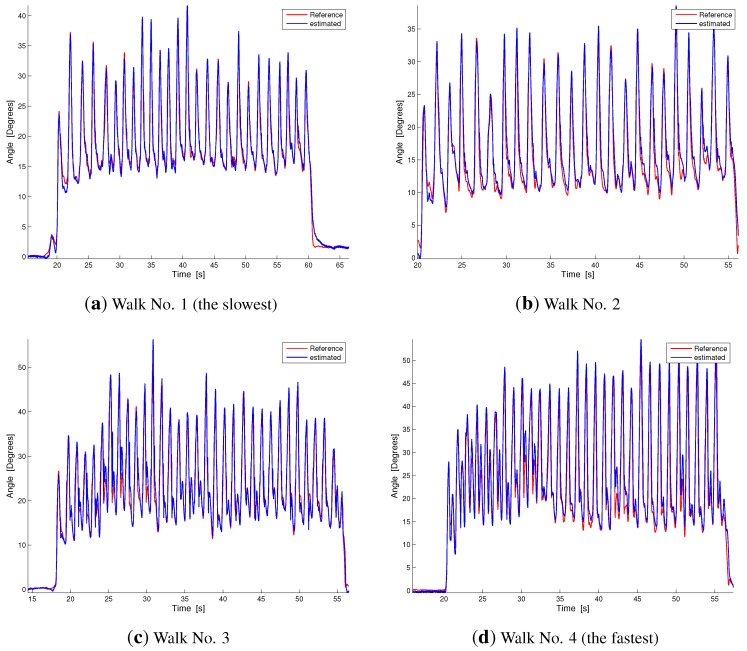
Dynamic comparison between our estimation technique and the reference measurement during walking at different velocities. Velocities increase from (**a**) to (**d**). The blue line represents our estimation, while the red line is the reference measurement.

Figure 5 compares the results of our estimation technique and the reference measurement system in two representative plots taken from the slow and fast monopodalic flexion trials (Figure 5a,b). Figure 6 shows the same comparison for representative plots of the four walking conditions ordered from the slowest (Figure 6a) to the fastest (Figure 6d). As can be observed from Figure 5 and Figure 6, our estimation technique shows a reliable performance and a good capability to follow the knee movement, both in slow and fast tasks.

For the six trials considered (slow flexion, fast flexion and the four walking conditions with increasing speed), we calculated the deviation between the estimation performed by the proposed technique applied to the hybrid system ($\theta_{hy}$) and the reference measurement ($\theta_{r}$ extracted from the IMUs) in terms of root mean square error (RMSE). Likewise, we evaluated the RMSE of the KPF goniometer ($\theta_{g}$) and accelerometer ($\theta_{a}$) estimations against the reference measurement system. The relations describing the RMSE deviations are reported in the following equations:

(28) $$RMSE_{m}^{i,j}={∥\theta_{r}^{i,j}-\theta_{m}^{i,j}∥}_{2}=\frac{1}{K^{i,j}}\sqrt{\sum_{k=1}^{K^{i,j}}{\left(\theta_{r}^{i,j}\left(k\right)-\theta_{m}^{i,j}\left(k\right)\right)}^{2}}$$

(29) $$RMSE_{m}^{i}=\frac{1}{N}\sum_{j=1}^{N}RMSE_{m}^{i,j}$$

where i represents the *i*-th trial (i=1 slow flexion, i=2 fast flexion, i=3 the slowest walking, i=6 the fastest walking), *j* denotes different subjects, N=5 is the number of subjects, $K^{i,j}$ is the number of samples of the *i*-th trial for the *j*-th subject and the suffix *m* indicates the different measurement systems. In particular, m=hy holds for the hybrid system, m=g for the goniometer, and m=acc refers to the accelerometers. Data obtained from the statistic Equation (29) are reported in Table 1 with the average value *μ* and the standard deviation *σ* computed over the six trials (last two columns of Table 1).

**Table 1:** Root mean square errors (RMSEs) obtained in the six trials for the various estimation methods. The first row ($RMSE_{hy}$) reports the errors of the hybrid system by applying the fusion technique described in this paper. The second and third rows show the textile goniometer ($RMSE_{g}$) and accelerometer ($RMSE_{acc}$) errors. The last two columns report the mean and standard deviation of the RMSE across the trials for the different measurement systems.

	Slow Flexion	Fast Flexion	Walking No. 1	Walking No. 2	Walking No. 3	Walking No. 4	Average	Standard
			(Slowest)			(Fastest)	Value *μ*	Deviation *σ*
$RMSE_{hy}$	0.97${}^{\circ }$	3.50${}^{\circ }$	1.07${}^{\circ }$	1.6${}^{\circ }$	2.1${}^{\circ }$	2.5${}^{\circ }$	1.96${}^{\circ }$	0.96${}^{\circ }$
$RMSE_{g}$	5.12${}^{\circ }$	4.60${}^{\circ }$	5.40${}^{\circ }$	4.6${}^{\circ }$	5.5${}^{\circ }$	5.7${}^{\circ }$	5.15${}^{\circ }$	0.47${}^{\circ }$
$RMSE_{acc}$	1.48${}^{\circ }$	10${}^{\circ }$	5.80${}^{\circ }$	6.7${}^{\circ }$	7.1${}^{\circ }$	8.2${}^{\circ }$	6.55${}^{\circ }$	2.87${}^{\circ }$

## 5. Discussion

Our hybrid system’s reliable performance is confirmed by the results reported in the first row of Table 1. The mean and standard deviations of the RMSE are 1.96 and 0.96, respectively. The minimum deviation was detected in the slow flexion trial ($RMSE_{hy}^{1}=0.97^{\circ }$), while the maximum error was in the fast flexion experiment ($RMSE_{hy}^{2}=3.5^{\circ }$). The deviations in the walking tasks range from $1.07^{\circ }$ for the slowest task to $2.5^{\circ }$ for the fastest. The first row of Table 1 and Figure 5 and Figure 6 highlight that in the hybrid system, RMSE increases as the speed of movement increases. In addition, fast walking trials show a lower error than fast flexion trials in monopodalic standing (as can be seen by comparing Figure 5b and Figure 6d and from the related RMSEs in Table 1). This is reinforced by the fact that the maximum error was in the fast knee flexion-extension trial reported in Figure 5b. This issue can be well explained by considering the estimation technique described in Section 3.1. Indeed, in the fast flexion trial, performed at maximum velocity in the range [0–$90^{\circ }]$, the rotational acceleration measured by the shank accelerometer is far from being small with respect to gravity and translational contributions. The smaller error in the walking trials may be explained considering that, during walking, even at high speeds, the rotational acceleration is sufficiently small. A comparison with the current literature reveals that joint angle measurements with commercial solid state electrogoniometers, widely used for the ambulatory evaluation of the range of motion and movement frequency/velocity/acceleration of the joints for both clinical and occupational evaluations [47,48], shows errors greater than two degrees, with a strong dependence on the sensor positioning and on the cross-talk between joints [49]. The reduced sensing systems, described in Section 1 [23,32,33,34,35,36,37] and based on two accelerometer/gyroscope pairs, achieved average RMSEs in the range $[2^{\circ }$–$7^{\circ }]$.

**Figure 7:**
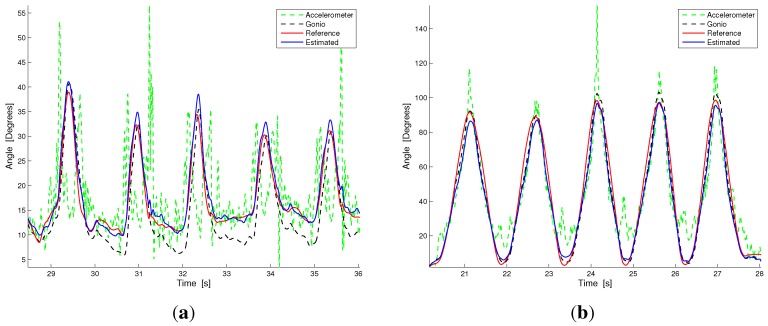
Signal comparison between the angle reconstruction by the accelerometers (green dotted line), the goniometer (black dotted line), the hybrid system (accelerometer + goniometer, blue solid line) and the reference measurement. (**a**) Walking; (**b**) Fast flexion.

We performed a further analysis to quantify the improvement introduced by the sensor fusion technique with respect to the single sensing subsystems (*i.e*., accelerometers and goniometer applied individually without fusing the information). The RMSEs related to the goniometer estimation of the knee flexion-extension angle are reported in the second row of Table 1. The mean and standard deviations of the RMSE are $5.15^{\circ }$ and $0.47^{\circ }$ (in accordance with the results we obtained in a previous work on textile goniometer angular measurement [29]). In addition, given how the KPF goniometer works, the RMSE variability across the trials, performed at different velocities, is quite low. The accelerometer RMSEs are reported in the third row of Table 1. The mean and standard deviation of the error hold $6.55^{\circ }$ and $2.87^{\circ }$, and as expected, there is a strong dependence on the execution velocity of the trial (from $1.48^{\circ }$ for the slow flexion, to $10^{\circ }$ for fast flexion). The last two columns of Table 1 highlight that the error introduced by the hybrid system ($\mu =1.96^{\circ }$, $\sigma =0.96^{\circ }$), evaluated in terms of RMSE, is considerably smaller than both the average errors of the goniometer ($\mu =5.15^{\circ }$, $\sigma =0.47^{\circ }$) and the mean of the accelerometers ($\mu =6.55^{\circ }$, $\sigma =2.87^{\circ }$). In addition, the error of the hybrid system is smaller than the goniometer and accelerometer systems in each of the tasks considered. Figure 7 highlights the angle estimation *vs*. the reference for the fusion method made on the hybrid system and the corresponding estimation made with the goniometer and accelerometers individually (representative plots of monopodalic flexion and walking tasks).

In the accelerometer signal, the errors are due to the acceleration spikes that are present when changing movement direction and when interacting with the environment (e.g., when the foot hits the floor in walking) and, thus, justify the large mean RMSE and the related standard deviation. Conversely, the goniometer signal is affected by errors due to the limitation introduced by the use of the nominal calibration parameters and slight hysteresis, which produced the error reported in Table 1. In conclusion, our fusion method compensates for these errors and consistently improves the accuracy of the estimation with respect to the single sensing subsystems taken individually.

One limitation of the current study is that the hybrid system and the fusion technique were evaluated against inertial measurement units. It is well known that the golden standard in biomechanics is marker-based optical tracking. Optical motion capture systems based on external markers have been extensively used in gait analysis and have shown, for the knee flexion-extension angle, RMSE deviations in the order of $2^{\circ }$ in comparison with bone fixed markers [50]. However, we decided to evaluate our system with respect to the IMU reference, so that we would be able to track the subject’s activity in an unconstrained environment (free walking tasks whose duration was about 40 seconds). In any case, we have now begun evaluating the hybrid system during the daily life activity of subjects where the IMU reference represents the only valid option. On the other hand, as demonstrated in [15], the estimation of the knee joint angle through the IMU-based system we used has shown reliable performance (errors in the order of two degrees).

A second limitation, which we are currently addressing, is that only one degree of freedom can be monitored per joint. We did not consider the rotation of the knee on the horizontal plane, since KPF goniometers are not influenced by twisting. From [29], where the complete form of relationship Equation (1) is reported in Taylor expansion form, it is possible to verify that there is no second order derivative term and, consequently, that the goniometer output ΔR does not depend on its torsion. To detect knee torsion, another goniometer is needed, which is placed in a crossed position, with one extremity on the thigh and the other one on the shank. In our solution, the global curvature of the second goniometer is related to knee torsion. We are currently working on the modeling of this sensing configuration and on the relative calibration strategy.

## 6. Conclusions

We have developed and conducted a preliminary evaluation of a new sensing concept aimed at unobtrusive and ambulatory human motion monitoring that combines e-textile and accelerometer sensors. Our hybrid system is based on textile goniometers and low cost accelerometers whose outputs are fused through a Kalman-based algorithm specifically developed for this work. The fusion technique continuously adjusts the goniometer electromechanical parameters using the information taken from the triaxial accelerometers. The method was applied in the measurement of the flexion-extension angle of the knee through the dedicated setup described in this paper. An important feature of the proposed method is that the initial position and orientation of the accelerometer with respect to the joint segments are unknown. Our calibration procedure aligns the accelerometer frames as required by the fusion technique. This procedure is easy to perform and is compatible with the ambulatory context that we are targeting. The estimation performance was compared to a reference measurement system during different motion tasks (monopodalic knee flexion and walking activity at different velocities). Our method showed a reliable performance and good capabilities in following dynamic knee movements (mean and standard deviation of the RMSE equal to 1.96 and 0.96 degrees). In addition, the method showed a notable improvement in the angle reconstruction compared to the estimations derived from the goniometer (RMSE: $\mu =5.15^{\circ }$, $\sigma =0.47^{\circ }$) and the accelerometers (RMSE: $\mu =6.55^{\circ }$, $\sigma =2.87^{\circ }$) considered separately. The next step in this research will be the generalization of the method to the monitoring of multi-degree of freedom joints.
